# Recruitment strategies and lessons learned from a large genetic study of African Americans

**DOI:** 10.1371/journal.pgph.0000416

**Published:** 2022-08-05

**Authors:** Rebecca J. Salowe, Roy Lee, Selam Zenebe-Gete, Marquis Vaughn, Harini V. Gudiseva, Maxwell Pistilli, Ava Kikut, Emily Becker, David W. Collins, Jie He, Sayaka Merriam, Kristen Mulvihill, Nora Laberee, Sara Lomax-Reese, Windell Murphy, Jeffrey Henderer, Venkata R. M. Chavali, Qi N. Cui, Ahmara G. Ross, Victoria Addis, Prithvi S. Sankar, Eydie Miller-Ellis, Maureen G. Maguire, Joan M. O’Brien

**Affiliations:** 1 Scheie Eye Institute, University of Pennsylvania, Philadelphia, PA, United States of America; 2 WURD Radio, Philadelphia, PA, United States of America; 3 Windell Murphy, MD, Philadelphia, PA, United States of America; 4 Lewis Katz School of Medicine at Temple University, Philadelphia, PA, United States of America; The Hospital for Sick Children, CANADA

## Abstract

Genetic studies must enroll large numbers of participants to obtain adequate statistical power. Data are needed on how researchers can best use limited financial and practical resources to achieve these targets, especially in under-represented populations. This paper provides a retrospective analysis of the recruitment strategies for a large glaucoma genetics study in African Americans. The Primary Open-Angle African American Glaucoma Genetics study enrolled 10,192 African American subjects from the Philadelphia region. Major recruitment approaches included clinic enrollment from University of Pennsylvania (UPenn) sites, clinic enrollment from external sites, sampling of Penn Medicine Biobank (PMBB), and community outreach. We calculated the enrollment yield, cost per subject, and seasonal trends of these approaches. The majority (65%) of subject were enrolled from UPenn sites with an average cost of $133/subject. Over time, monthly case enrollment declined as the pool of eligible subjects was depleted. Expanding to external sites boosted case numbers ($129/subject) and the biobank provided additional controls at low cost ($5/subject), in large part due to the generosity of PMBB providing samples free of cost. Community outreach was costly with low return on enrollment ($978/subject for 220 subjects). Summer months (Jun-Aug) produced the highest recruitment yields (p<0.001). Genetic studies will benefit from a multi-pronged and culturally sensitive recruitment approach. In our experience, the biobank was most cost-effective for control enrollment, while recruitment from clinics (including expansion to new sites) was necessary to recruit fully phenotyped cases.

## Introduction

The power of a genome-wide association study (GWAS) to detect causal variants is largely determined by the size of its enrolled cohort [1, 2]. An increase in sample size can have a greater effect on statistical power than any other variable, including number or coverage of single nucleotide polymorphisms (SNPs) on a genotyping chip [2]. In order to reach the high enrollment numbers needed for well-powered genetic studies, it is important to understand the unique challenges associated with recruitment in genetic research. Unlike other clinical studies, genetic research involves the collection of DNA, introducing concerns such as confidentiality, misuse of information, and genetic or racial discrimination [3, 4].

African American individuals have been historically under-represented in genetic research [5, 6]. As of 2016, only 3% of participants in GWAS were of African descent [5]. Both the decreased likelihood of these individuals to consent to genetic studies [7], as well as the lack of outreach to the African American community by researchers [8], likely contribute to this disparity. Some African American individuals are distrustful of genetic researchers due to past abuses, such as the infamous Tuskegee Syphilis Study [9, 10]. Concern over future use of DNA samples [9, 11] and cultural differences between investigators and patients [12, 13] have also been cited as barriers to recruitment in the African American population. Many recruitment tactics, which were tailored by and to white individuals, may not take these specific barriers into account [14]. Additionally, it is possible that some researchers may be reluctant to focus limited resources on a population that they perceive as “difficult to recruit.” In fact, one study showed that “not being asked” was the main reason that African Americans had not previously participated in genetic research [15].

When recruiting African American individuals for genetic studies, researchers have a responsibility to understand and address these complex issues. As a whole, enrollment strategies should be culturally sensitive and seek to build trust in potential participants. Studies have consistently shown that involvement of African American researchers in the recruitment process [16, 17], option to use a non-invasive method of DNA collection method (rather than blood) [18], and strong relationships with physicians [19, 20] contribute to this population’s comfort and willingness to enroll in genetic research. The involvement of African American churches and community leaders also helps to build trust and encourage enrollment [20, 21] The opportunity to receive personal genomic risk results also appears to be a powerful incentive for many African American individuals [22, 23] These approaches are critical to integrate into genetic studies of African Americans whenever possible.

To our knowledge, no large genetic study of African American individuals has provided a retrospective analysis of the successes and failures of their recruitment approaches. Some studies report the results of specific efforts, such as projects evaluating the efficacy of media advertisements [24] or considering potential disadvantages of using biobank samples [25]. However, large-scale questions such as which enrollment sites will be most cost-effective, and whether or not to incorporate methods such as biobank sampling, community outreach, or other advertising efforts, are also important to address in the study design phase. A detailed analysis of these factors could help researchers better anticipate costs, make informed decisions on enrollment sites and methods, maximize enrollment numbers and statistical power, and support additional studies of African American populations.

This study provides a retrospective analysis of the recruitment approaches for a large glaucoma genetics study in African Americans. The Primary Open-Angle African American Glaucoma Genetics (POAAGG) study investigates the genetic risk factors for primary open-angle glaucoma (POAG) in African Americans, who are disproportionately affected by this familial disease [26]. This study enrolled more than 10,000 African Americans from the Philadelphia region by employing four major recruitment strategies. Here, we assess the enrollment yield, cost, and advantages and disadvantages of each recruitment method, with the goal of providing useful information to guide future studies in African Americans.

## Methods

### Ethics statement

The study protocol and consent statement were approved by the University of Pennsylvania institutional review board (IRB). This research adhered to the tenets of the Declaration of Helsinki. All subjects signed a written informed consent statement.

### Study population

The POAAGG study has a case-control design. Enrollment for the study began in July 2010 and concluded in March 2019. The study population consists of self-identified Blacks (African Americans, African descent, or African Caribbean), 35 years or older, identified from the Philadelphia region. Inclusion and exclusion criteria are described elsewhere [26].

Each enrolled subject was classified by a glaucoma specialist or ophthalmologist as a case, control, or suspect based on detailed criteria [26]. In brief, cases were defined as having an open iridocorneal angle and characteristic optic nerve defects with corresponding visual field loss, while controls were patients seen in regularly scheduled ophthalmology appointments without a glaucoma diagnosis or confounding ocular conditions [26]. Suspects were defined as individuals with risk factors that indicate an increased likelihood of developing POAG, but without optic nerve damage and/or visual field defects.

The initial enrollment goal of the POAAGG study was 7765 subjects (3000 cases and 4765 controls). However, as the study proceeded, we sought to exceed that goal in order to maximize statistical power in the GWAS. Subjects were recruited to the POAAGG study using four main strategies, as detailed below.

### Strategy #1: Clinic enrollment at University of Pennsylvania (UPenn) sites

Enrollment for the POAAGG study began in July 2010 at three UPenn sites: Scheie Eye Institute, Perelman School of Medicine, and Mercy Fitzgerald Hospital. A fourth site (Philadelphia VA Medical Center) was added in March 2018. UPenn-certified Clinical Research Coordinators (CRCs) approached eligible patients during regularly scheduled ophthalmology appointments. Each research subject received a clinical examination, including an onsite interview and ophthalmic exam. Examination data were recorded on case report forms and entered directly into the REDCap (Research Electronic Data Capture) database [27, 28]. These data were later supplemented with retrospective ophthalmic and systemic data extracted from electronic medical records (UPenn EPIC and Merge PACS databases). All subjects signed an informed consent form and provided a genomic DNA sample, which was collected from peripheral blood (July 2010-November 2014) or saliva (November 2014-present) by the CRC, before being sent to our laboratory for DNA extraction.

### Strategy #2: Clinic enrollment at external sites

Clinic enrollment also took place at two ophthalmology clinics outside of UPenn: the private practice of a Scheie Eye Institute alumnus (Windell Murphy, MD) and the Ophthalmology Department at the Lewis Katz School of Medicine at Temple University (Jeffrey Henderer, MD). A subset of subjects (n = 140) were enrolled from Temple University from May 2011 to February 2012, before pausing to focus on UPenn sites. Enrollment began in May 2015 at Windell Murphy, MD and re-started at Temple in July 2015, respectively. CRCs from the POAAGG study traveled to these sites on pre-scheduled dates and approached eligible potential subjects in the clinic. Enrollment procedures at these sites were identical to those at UPenn sites. All participating physicians were trained on the POAAGG classifications for cases, controls, and suspects. Phenotypic information from subjects was migrated to the REDCap database, following IRB research protocol data sharing policies.

### Strategy #3: Penn Medicine Biobank

The Penn Medicine Biobank (PMBB) has enrolled approximately 60,000 subjects to date, of whom nearly 25% are African American. Participation in the biobank involves a blood draw for DNA extraction, tissue sampling (if applicable), and a questionnaire. All subjects in the biobank have consented for their DNA to be used in research studies at UPenn.

The POAAGG study obtained a list of potential eligible subjects from the biobank and mailed each individual a letter, providing the opportunity to opt-out of our study. A small subset of subjects who had already enrolled in the POAAGG study were excluded. In September 2015, PMBB provided de-identified aliquots of DNA from 2073 eligible African American subjects, along with information on gender and ICD-9 codes.

Our team then attempted to obtain phenotypic information on these 2073 individuals. A brochure was mailed to PMBB subjects that explained the POAAGG study and provided a tear-off page to mail back with glaucoma status and family history information; a total of 129 individuals mailed back this tear-off page. Additionally, a total of 583 subjects had electronic medical records from a prior ophthalmology appointment at UPenn, which were carefully reviewed by a glaucoma specialist to provide more definitive classifications as case, control, or suspect.

### Strategy #4: Community outreach

We provided free glaucoma screenings to African Americans throughout the course of the study, with eligible individuals offered an opportunity to enroll in the POAAGG study. For all approaches, our study team of CRCs and glaucoma specialists had strong African American representation. Our CRC team has typically been approximately 75% individuals of color (though can shift year to year with new hires), and our Community Outreach Coordinator is an African American woman with strong ties to the Philadelphia community. Of the five glaucoma specialists contributing to this study, four are individuals of color (and two self-identify as African American). The glaucoma service is directed by an African American woman.

Outreach activities fell into three main categories, described in detail below.

1. Community Outreach Events: Beginning in August 2014, our team provided free glaucoma screenings at outreach events throughout the Philadelphia community on weekends. Events were conducted in North or West Philadelphia at sites within a 1–5 mile radius of the Scheie Eye Institute; these areas have high levels of poverty and low access to healthcare. Our Community Outreach Coordinator approached churches, community centers, and retirement homes in these areas to see if they would be willing to partner with us for an outreach event. The events were frequently publicized through partnerships with African American community leaders and pastors.

We transferred all necessary screening equipment to outreach sites using a mobile van, driven by a CRC (purchased using funds from a UPenn Hospital Board of Women Visitors grant). The majority of screening devices were borrowed from the UPenn Ophthalmology Department. An exception was the lease of a Cirrus HD-Optic Coherence Tomography (OCT) device (Carl Zeiss Meditec, Inc., Dublin, CA). Each community outreach attendee received an eye screening by a glaucoma specialist, regardless of eligibility for the study. Individuals eligible for the POAAGG study were offered the opportunity to enroll, and if accepted, proceeded through the normal enrollment process, including DNA collection and classification as a case, control, or suspect by a glaucoma specialist. These screenings were also used to detect and refer systemic conditions with ocular manifestations, such as diabetes and hypertension, and to provide trainings and giveaways of low vision devices. We also offered free lunch at outreach events.

We did not have a target enrollment goal for outreach events. All events were opened to any interested community members. We ensured that a full team of glaucoma specialists and CRCs were present at each event, so we could accommodate all patients.

2. In-House Screenings: Beginning in October 2014, our team provided free glaucoma screenings to African American individuals in a private exam room in the Scheie Eye Institute. We publicized these screenings primarily through a series of advertisements in the local subway (SEPTA). Interested community members were encouraged to call our team to schedule a free glaucoma screening without usual wait times. Eligible individuals were offered the opportunity to enroll in the POAAGG study and were classified as a case, control, or suspect by a glaucoma specialist.
3. Multimedia Campaign: From January 2018 to March 2019, our team conducted a multimedia messaging campaign throughout Philadelphia [10, 29]. The goals of this campaign were to increase awareness of glaucoma risk in the African American community, to provide free glaucoma screenings to individuals who may not have access to healthcare providers or insurance, and to enroll eligible individuals in the POAAGG study. The campaign involved multiple messaging approaches, with the majority arising from a partnership with WURD Radio (Black-owned health radio station in Philadelphia), including commercials, physician interviews, patient testimonials, show sponsorship, and WURD-sponsored health fairs. Other messaging approaches included written materials (study postcards, outreach flyers) and digital materials (social media, study website). We offered potential subjects the opportunity to receive their screening at an upcoming outreach event or private in-house screening. We used the TrialX iConnect recruitment tracking and analytics system to track the yield of each messaging approach (iConnect Patient Recruitment Management System, Version 3.0, TrialX Inc New York, NY 10016) [29].

### Cost analysis

To calculate the costs associated with each recruitment method, we categorized expenses into several categories, as detailed below. For costs that fluctuated over the course of the study due to inflation or other factors, we used the original cost in the below analyses.

1. Blood Collection: From July 2010 to November 2014, the CRC collected a blood sample from each enrolled subject, with average cost of $2.82/subject. This cost covers the materials required for a blood draw (IV starter kit, needle holder, tubes, needles), which we purchased in bulk. Each CRC was trained in phlebotomy at UPenn, so no additional phlebotomist time included was required.
2. Saliva Collection: From December 2014 to present, we collected a saliva sample from each enrolled subject, which cost $17.25/subject. This cost reflects the saliva collection kit used for each subject, which we purchased in bulk. The cost of DNA extraction for blood versus saliva was comparable and was not included in recruitment costs.
3. Gift Card: Each subject was given a $10 gift card after completing the enrollment process.
4. Personnel Time: The calculation of non-physician personnel time varied by strategy. We made the below assumptions:
  ■ UPenn Sites/External Sites: We estimated the number of CRCs actively enrolling subjects each month by checking the history of user activity in REDCap. Throughout these analyses, one CRC denotes one full-time employee. These estimates were adjusted to account for CRCs being away or focused on a non-enrollment related project (i.e. if a CRC was only active for two weeks in a given month, he or she would count as 0.5 for that month). Total cost of personnel time for clinic enrollment was calculated for each month, using a rate of $15/hour. This total was then divided among individual sites. For example, if 36 of 39 subjects (92%) were enrolled at Scheie in July 2010, we assumed that 92% of CRC time was spent at Scheie.
  ■ Penn Medicine Biobank: We asked the relevant personnel to track and record the number of hours spent reviewing the returned brochures and the electronic medical records of 583 subjects to determine subject classification.
  ■ Outreach events: We estimated that each event involved approximately four hours of preparation (four CRCs), five hours at the event (eight CRCs), and three hours of subsequent follow-up (four CRCs). A total of 9 outreach events were conducted.
  ■ In-house screenings: We estimated that each screening involved approximately 2.5 hours (two CRCs).
  ■ Multimedia Campaign: We assumed that our Community Outreach Coordinator spent approximately 17 hours on subject phone calls, 13 hours on WURD-sponsored health fairs, and 8 hours on postcard distribution. Personnel time for screenings that arose from campaign inquiries was included in this category, using the same estimations as listed above.
5. Physician Time: Physician time at outreach events and in-house screenings was generously donated (i.e., conducted on weekends or in addition to normal clinic hours). For the campaign, the number of hours spent conducting WURD radio interviews and screening scheduled individuals was calculated, as this was conducted during normal weekday hours.
6. Transportation: For each external site, the number of CRCs enrolling at that site, as well as the total number of days spent enrolling at that site, was estimated and multiplied by the round-trip cost of transport ($4 on subway). Transportation costs for outreach events included the van purchase, annual parking fee, and fuel.
7. Equipment: The majority of screening equipment used for this study was already present at Penn and external sites and was not included in the cost analysis. Exceptions include the lease of an OCT device designated for study subjects and specific equipment needed for outreach events (i.e., privacy screens, tables, etc.).
8. Advertising: The cost of printing written materials such as postcards and outreach flyers was included, as well as the price paid to platforms used for study promotion, including SEPTA and WURD radio.
9. Other: Miscellaneous costs included opt-out cards and brochures for biobank subjects, and parking passes and cab vouchers for campaign subjects.

## Results

The POAAGG study enrolled a total of 10,192 subjects, including 2883 cases, 5800 controls, and 1509 suspects (Table 1). The majority (65%) of subjects were enrolled from UPenn sites, followed by PMBB (20%), external sites (13%), and community outreach (2%). Cases (97%) were predominantly recruited from ophthalmology clinics at UPenn and external sites. For community outreach efforts (n = 220), recruitments arose from outreach events (n = 114), in-house screenings (n = 46), and the multimedia campaign (n = 60).

**Table 1:** Total enrollment for POAAGG study from 07/2010-03/2019 by recruitment strategy.

Approach	Case	Control	Suspect	Total	Cost per Subject
**1. Penn Sites**	2285 (79%)	3325 (57%)	1000 (66%)	6610 (65%)	$133
**2. External Sites**	511 (18%)	408 (7%)	370 (25%)	1289 (13%)	$129
**3. Biobank (PMBB)**	31 (1%)	1954 (34%)	88 (6%)	2073 (20%)	$5
**4. Community Outreach**	56 (2%)	113 (2%)	51 (3%)	220 (2%)	$978
***Total***	***2883***	***5800***	***1509***	***10*,*192***	

UPenn sites included ophthalmology clinics at the Scheie Eye Institute, Perelman School of Medicine, Mercy Fitzgerald Hospital, and Philadelphia VA Medical Center. External sites included the private practice of Windell Murphy, MD and the ophthalmology clinic at the Lewis Katz School of Medicine at Temple University. The biobank refers to eligible subjects from the Penn Medicine Biobank. Community outreach refers to all subjects recruited through outreach events, in-house screenings, or a multimedia campaign. A small number of patients with unknown classification as case, control, or suspect were excluded from this table.

In terms of cost per subject enrolled, PMBB had the lowest expense for the POAAGG study at $5/subject (Table 2), because most recruitment cost was borne by the biobank. UPenn and external sites had similar costs at $133/subject and $129/subject, respectively. Community outreach was associated with the highest costs, averaging $978/subject. The largest contributor to recruitment costs was non-physician personnel time (70% of total costs).

**Table 2:** Breakdown of major recruitment costs for the POAAGG study.

	Blood Collection	Saliva Collection	Gift Card	Personnel Time	Physician Time	Transport-ation	Equipment	Advertising	Other	# Enrolled	Total Cost	Cost per Subject
**Penn Sites**	$7947	$65,412	$66,100	$741,546	$0	$779	$0	$0	$0	6610	$881,783	$133
**External Sites**	$395	$19,820	$12,890	$129,715	$0	$2871	$0	$0	$0	1289	$165,691	$129
**Biobank (PMBB)**	N/A	N/A	N/A	$4668	N/A	N/A	N/A	N/A	$4881	2073	$9549	$5
**Community Outreach**	$82	$3329	$2220	$17,820	$1987	$35,612	$100,960	$52,580	$607	220	$215,197	$978
**Total Cost**	**$8423 (0.7%)**	**$88,562 (7.0%)**	**$81,210 (6.4%)**	**$893,748 (70.3%)**	**$1987 (0.2%)**	**$39,261 (3.1%)**	**$100,960 (7.9%)**	**$52,580 (4.1%)**	**$5488 (0.4%)**	**10,192**	**$1,272,219**	

*Blood Collection: $2.82 /subject

†Saliva Collection: $17.25/subject

‡Gift Cards: $10/subject.

**Fig 1:**
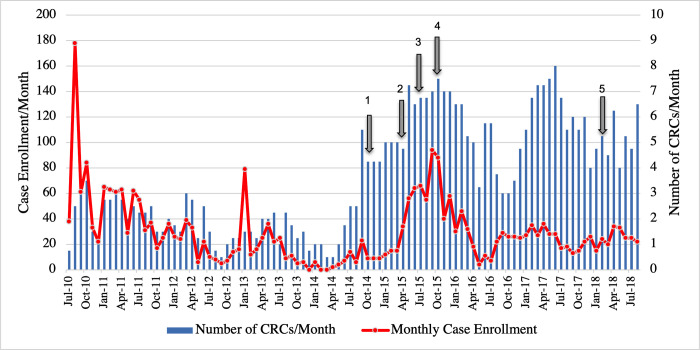
Monthly enrollment of glaucoma cases for the Primary Open-Angle African American Glaucoma Genetics study. Monthly case enrollment declined over time, despite increased number of Clinical Research Coordinators (CRCs) to enroll subjects. The arrows denote five significant shifts in the enrollment process: (1) November 2014: Switch from blood samples to saliva samples for DNA collection, (2) May 2015: Addition of first external site (Windell Murphy, MD), (3) July 2015: Re-started enrollment at second external site (Temple University), (4) September 2015: Addition of samples from the Penn Medicine Biobank, and (5) March 2018: Addition of a fourth UPenn site (VA Hospital). Monthly enrollment is shown from 07/2010-07/2018, as after this time point, enrollment was intentionally slowed as CRC efforts were needed for other efforts such as phenotype collection or database management.

Monthly enrollment rates varied over the course of the POAAGG study, particularly among cases. Even as the number of CRCs employed rose, case enrollment began to drop over subsequent months, as the original pool of eligible individuals was depleted by enrollment into the study (Fig 1). The expansion to two external sites in 2015 helped to reach a new population of potential subjects. This expansion, coupled with the hiring of additional CRCs, increased monthly enrollment for roughly one year at similar cost, before these sites saw similar declines (Fig 1).

**Fig 2:**
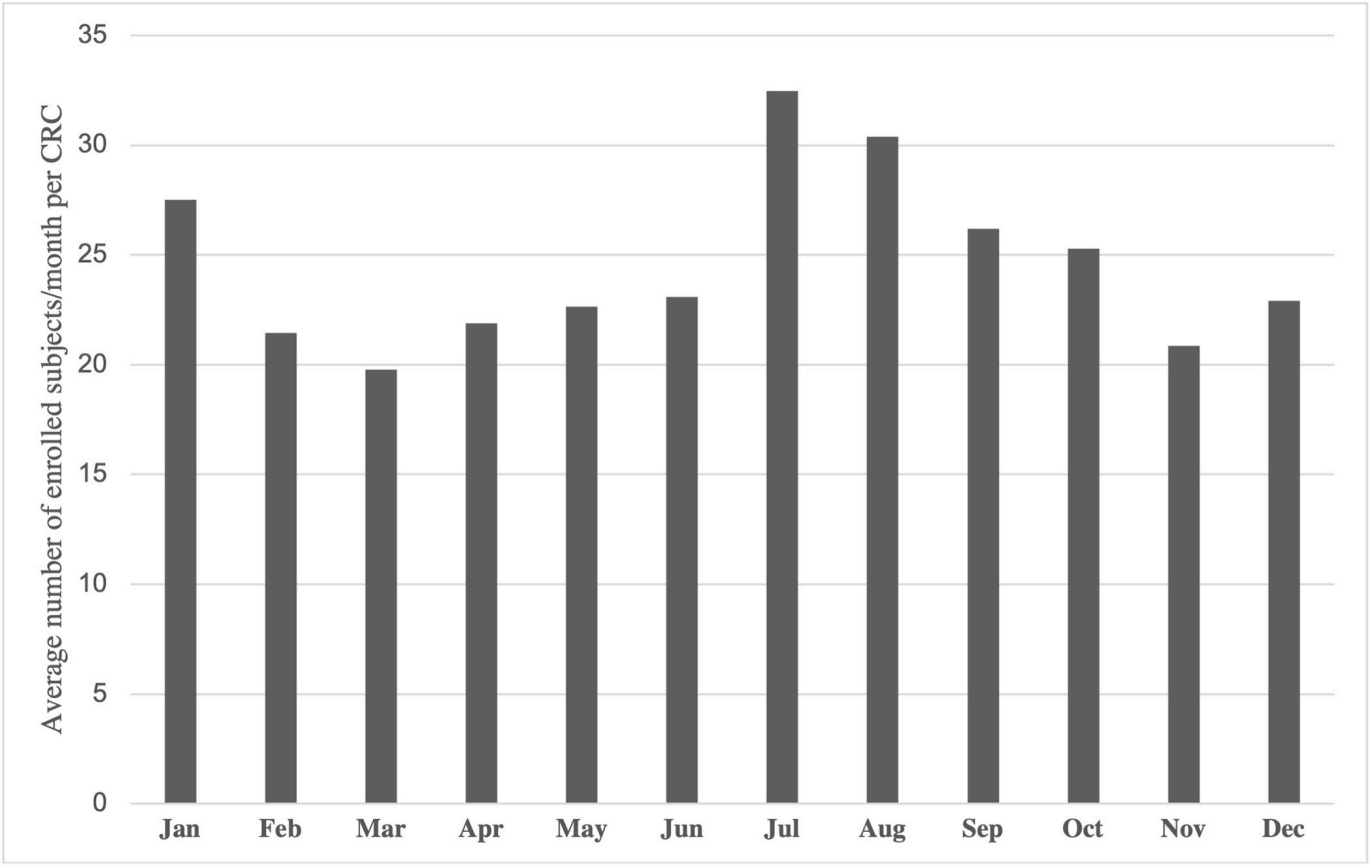
Seasonal trends in recruitment in the POAAGG study, displayed as average number of enrolled subjects/month per Clinical Research Coordinator (CRC). The months of July and August saw the highest enrollment yield, while spring months showed the lowest yield. Recruitment from the biobank is excluded from this figure. Data from 07/2010-07/2018 is included, as after this time point, enrollment was intentionally slowed as personnel efforts were needed for other efforts such as phenotype collection or database management.

When examining seasonal trends, the summer months (June to August) showed the highest recruitment yield compared to other seasons, with an average of 29 subjects/month enrolled per CRC (p<0.001, Fig 2). The spring months had the lowest enrollment yield (average 21 subjects/month per CRC), while autumn and winter months showed slightly higher numbers (average 24 subjects/month per CRC).

## Discussion

We conducted a retrospective analysis of the recruitment strategies employed by a large genetics study in African Americans. We found that traditional clinic enrollment was very effective for recruitment in early years, but declined over time as the pool of eligible subjects was depleted by our own successful enrollment efforts. After this drop-off, biobanks offered a rapid increase in control numbers at low cost, while expansion to external sites was necessary to continue to recruit phenotyped cases.

At the start of our study, we focused exclusively on enrollment at ophthalmology clinics at UPenn to meet the initial goal of 7765 enrolled subjects. We recruited 6610 subjects from these sites, including 79% of total cases (n = 2285). In terms of cost, we spent roughly $133/subject, which mostly arose from non-physician personnel time. However, after several years, we began to experience stagnation in case enrollment. The influx of new glaucoma patients to UPenn was not large enough to counteract this effect. The decrease in enrollment efficiency also began to affect costs, as more CRCs were needed to recruit fewer subjects (i.e. costs for Penn sites were $123/subject before May 2015 and $146/subject after May 2015). This transition prompted us to re-strategize and consider other methods, including expansion to external sites.

Expansion to external ophthalmology clinics in the Philadelphia region re-invigorated our case numbers without much added expense. We sought out ophthalmology clinics with fellowship-trained glaucoma specialists who served a sizable African American population. These ophthalmologists generously opened their clinics to our study free of charge, so costs remained very similar to enrollment from UPenn clinics. Recruitment from these sites also allowed our cohort to remain a single-city study, limiting genetic heterogeneity [30] and facilitating detailed phenotyping with an ability to re-contact subjects. However, case enrollment at external sites eventually slowed due to the same depletion problem as UPenn, leading us to decrease the frequency of recruitment from these sites.

The biobank strategy addressed a different need of the study, rapidly increasing control numbers at low cost to our study ($5/subject). Of note, PMBB researchers generously provided samples to our study free of charge; fees at other biobanks for biological samples can range widely. The main drawback to biobank recruitment was that 1490 out of 2073 subjects lacked phenotypic information outside of ICD-9 and ICD-10 codes, raising concern about the incidence of undiagnosed glaucoma and ultimately preventing their inclusion in the GWAS. Fortunately, we were able to obtain more detailed information on the remaining 583 subjects who had prior ophthalmology appointments at UPenn by searching their electronic health records. The majority of these 583 subjects were included in the GWAS and subsequent genetic analyses. The remaining 1490 subjects without ophthalmology records were excluded from genetic analyses out of an abundance of caution, as these individuals could represent subjects with undiagnosed glaucoma. Thus, though the biobank strategy rapidly increased enrollment numbers, only 28% of these individuals were ultimately included in the GWAS, representing a key weakness of this method. However, the addition of 583 subjects to genetic analyses was certainly not negligible, and the remaining 1490 subjects have been included in other clinical studies; thus, we would still recommend the biobank strategy to other studies due to its low cost, but advise researchers to be aware of the limitations posed by limited phenotypic information.

Community outreach allowed us to screen at-risk members of the African American community for glaucoma and other systemic diseases. Our goal was to reach individuals without access to healthcare providers or insurance; as a result, the majority of these individuals had not previously enrolled in the POAAGG study through other methods, such as clinic enrollment. In total, we screened 370 individuals for glaucoma, ultimately enrolling 220 eligible subjects (~13 subjects/event) at an average cost of $978/subject. A large contributor to the high costs was specialized screening equipment; for example, without the OCT machine lease ($19,392/year), the cost per subject would drop to $538/subject. We found that applying for smaller foundation grants for specific efforts (i.e. outreach bus) made these events more affordable and feasible. It is also important to note that the most important outcomes from outreach efforts cannot be quantified. Because glaucoma is insidious, more than 50% of patients are not aware of their diagnosis [31]. Our outreach efforts helped to identify cases of undiagnosed glaucoma, potentially preventing irreversible vision loss. Events with our community partners also helped to raise awareness of a disease that affects African Americans up to 15 times more severely than other ethnic groups [32]. Finally, these screenings also helped to capture and refer systemic conditions with ocular manifestations, such as diabetes, atherosclerosis, and hypertension.

There were significant differences in enrollment numbers between seasons, with the summer months yielding the highest recruitment. It is possible that patients felt greater ease or safety driving to appointments or taking public transportation in the mild weather. It is difficult to compare or generalize these trends to other studies, as results may vary based on the weather patterns of the recruitment region or the patient population being targeted. One multicenter clinical trial reported that enrollment accelerated in the spring and late fall [33]. Another found that intervention-based studies had peak registration around the New Year [34]. Others have cited transportation issues as the most critical non-medical recruitment concern, especially among individuals with lower incomes [35]. Giving special care to transportation access, especially in seasons of more unpredictable or hazardous weather, could help to counteract enrollment declines.

Finally, it is important to acknowledge that some contributors to recruitment cannot be measured or quantified. At every site, we strove to employ a framework of cultural humility that was sensitive to the needs of elderly African Americans. We additionally hired a study team with strong African American representation among both CRCs and glaucoma specialists, and consistently partnered with local community leaders. For our outreach efforts, we worked to schedule appointments around patient availability, to offer financial assistance with transportation, and to guide individuals towards insurance options when needed. When improvements in saliva stabilization technology became available in 2014, we switched from blood to saliva collection to increase patient comfort and ease of enrollment, despite the higher cost [18]. We believe that such a full-scale effort is needed to complement even the strongest recruitment strategy.

There are several limitations to this study. First, for the cost analysis, we used exact numbers whenever possible, but it was necessary to approximate other costs, such as personnel and physician time. Our study was also on the receiving end of much generosity, including physicians donating time for outreach activities, external investigators opening their clinics for recruitment, and PMBB providing samples free of charge. We were transparent about these specifics for our study, but fully recognize that these costs may not fully reflect or predict costs for research groups at different Universities. Second, we do not have information on the response rate for each approach, as information on individuals who declined enrollment was not recorded over the course of the study. We also acknowledge that the success of recruitment at different sites, or the decline of numbers over time, could be influenced by other factors that are not discussed in this paper. For example, the time and length of implementation for each recruitment approach could be a potential confounder. Finally, though a main goal of this retrospective study was to provide useful information to other genetic researchers, we recognize that our study was conducted on African American individuals living in Philadelphia, with and without glaucoma. Our results may not be fully generalizable to other cities, diseases, age groups, or racial and ethnic groups.

## Conclusions

This paper provides a retrospective analysis of the enrollment strategies of a large genetic study, which recruited more than 10,000 African American individuals from a single city. We present a transparent analysis of costs, enrollment yield, and seasonal trends of various approaches. Overall, we found that clinic enrollment was very effective in early years, but slowed over time as the pool of eligible subjects was depleted. After this drop-off, expansion to external sites allowed us to continue to recruit phenotyped cases, while biobanks offered a rapid increase in control numbers at low cost. Overall, we show that it is possible to recruit a large cohort for genetic studies by employing a multi-pronged approach and developing culturally sensitive methods in minority populations. This thorough report may help to inform future enrollment strategies for other studies, especially in the frequently understudied and overaffected Black population.

## Supporting information

S1 Data Contains all data that was used in tables, figures, and calculations in this paper. (XLSX)
